# Predicting Patient Satisfaction With Medications for Treating Opioid Use Disorder: Case Study Applying Natural Language Processing to Reviews of Methadone and Buprenorphine/Naloxone on Health-Related Social Media

**DOI:** 10.2196/37207

**Published:** 2023-01-23

**Authors:** Samaneh Omranian, Maryam Zolnoori, Ming Huang, Celeste Campos-Castillo, Susan McRoy

**Affiliations:** 1 Department of Electrical Engineering and Computer Science College of Engineering & Applied Science University of Wisconsin-Milwaukee Milwaukee, WI United States; 2 School of Nursing Columbia University New York, NY United States; 3 Department of Artificial Intelligence and Informatics Mayo Clinic Rochester, MN United States; 4 Department of Media and Information Michigan State University East Lansing, MI United States

**Keywords:** machine learning, online forums, text classification, topic modeling, MetaMap, drug review, opioid treatment, opioid use disorder, patient-generated text

## Abstract

**Background:**

Medication-assisted treatment (MAT) is an effective method for treating opioid use disorder (OUD), which combines behavioral therapies with one of three Food and Drug Administration–approved medications: methadone, buprenorphine, and naloxone. While MAT has been shown to be effective initially, there is a need for more information from the patient perspective about the satisfaction with medications. Existing research focuses on patient satisfaction with the entirety of the treatment, making it difficult to determine the unique role of medication and overlooking the views of those who may lack access to treatment due to being uninsured or concerns over stigma. Studies focusing on patients’ perspectives are also limited by the lack of scales that can efficiently collect self-reports across domains of concerns.

**Objective:**

A broad survey of patients’ viewpoints can be obtained through social media and drug review forums, which are then assessed using automated methods to discover factors associated with medication satisfaction. Because the text is unstructured, it may contain a mix of formal and informal language. The primary aim of this study was to use natural language processing methods on text posted on health-related social media to detect patients’ satisfaction with two well-studied OUD medications: methadone and buprenorphine/naloxone.

**Methods:**

We collected 4353 patient reviews of methadone and buprenorphine/naloxone from 2008 to 2021 posted on WebMD and Drugs.com. To build our predictive models for detecting patient satisfaction, we first employed different analyses to build four input feature sets using the vectorized text, topic models, duration of treatment, and biomedical concepts by applying MetaMap. We then developed six prediction models: logistic regression, Elastic Net, least absolute shrinkage and selection operator, random forest classifier, Ridge classifier, and extreme gradient boosting to predict patients’ satisfaction. Lastly, we compared the prediction models’ performance over different feature sets.

**Results:**

Topics discovered included oral sensation, side effects, insurance, and doctor visits. Biomedical concepts included symptoms, drugs, and illnesses. The F-score of the predictive models across all methods ranged from 89.9% to 90.8%. The Ridge classifier model, a regression-based method, outperformed the other models.

**Conclusions:**

Assessment of patients’ satisfaction with opioid dependency treatment medication can be predicted using automated text analysis. Adding biomedical concepts such as symptoms, drug name, and illness, along with the duration of treatment and topic models, had the most benefits for improving the prediction performance of the Elastic Net model compared to other models. Some of the factors associated with patient satisfaction overlap with domains covered in medication satisfaction scales (eg, side effects) and qualitative patient reports (eg, doctors’ visits), while others (insurance) are overlooked, thereby underscoring the value added from processing text on online health forums to better understand patient adherence.

## Introduction

### Opioid Use Disorder

The 2018 National Survey on Drug Use and Health estimated that 10.3 million people over 12 years old misused opioids, including 9.9 million individuals who misused prescribed pain relievers and 808,000 heroin users [1]. Long-term misuse of opioids and heroin affects the brain’s normal functionalities and results in opioid tolerance, dependence, or addiction ‎[2]. The Centers for Disease Control and Prevention (CDC) has preferred the term “opioid use disorder” (OUD) over “opioid abuse or dependence” owing to the set of behavioral, cognitive, and physiological symptoms after repeated substance use ‎[3]. In response to the opioid dependence crisis, the National Institute on Drug Abuse has invested in the implantation of science and patient care to increase access to medication-assisted treatment (MAT), which consists of medication and behavioral therapies to reduce OUD across health care and the justice system ‎[4]. MAT with opioid agnostic medications such as buprenorphine and methadone helps patients with OUD reduce relapse rates of quitting opioids; lowers illicit opioid use; and results in an overall reduction of the burden of opioid dependency on patients, caregivers, and the health care system ‎[5].

Based on the CDC reports, MAT is a practical, systematic approach that incorporates medications such as methadone, buprenorphine, or naloxone along with behavioral therapy to meet the needs of patients with OUD. Methadone, a full opioid agonist, has been the most generally recognized and well-researched among pharmacological and nonpharmacological treatments since its introduction in 1965 ‎[6]. Methadone provokes cells in the same way as illicit opioids but does not invoke the same cellular response that leads to dependence on the drug ‎[7]. Another well-tolerated MAT supervised by the medical profession is buprenorphine/naloxone, marketed under the brand name Suboxone ‎[8]. Buprenorphine is a partial opioid agonist that binds to the same opioid receptors as the opioid drugs in the brain, decreasing craving and withdrawal symptoms ‎[8]. Methadone was developed for oral applications, and buprenorphine/naloxone is formulated for sublingual applications ‎[9].

In a controlled comparative randomized study, Saxon et al ‎[10] assessed the retention rates of methadone and buprenorphine/naloxone in individuals with OUD (N=1269). The study found that 74% of patients taking methadone completed the 24-week treatment, while only 46% of patients taking buprenorphine/naloxone completed the 24-week treatment, suggesting that over the net of behavioral therapies and other client services offered, medications play a key role in experiences. These findings suggest that a methadone treatment course may produce a better retention rate (medication’s overall effectiveness) than a buprenorphine/naloxone treatment course, yet patients may prefer the latter when given a choice [11].

Various factors play into patient preferences and overall satisfaction with medication, including financial barriers, ease of use, and side effects, particularly withdrawal symptoms [12]. Unlike MAT, withdrawal symptoms appear immediately after opioid discontinuation or lowering of the dosage of opioids ‎[13,14]. Cicero et al [15] found that the fear of withdrawal symptoms is a compelling motivator to relapse a short time after OUD treatment. As a result, poor/nonadherence and treatment dropout are quite common in MAT ‎[16]. Bastiaans et al [17] reported elevated pulse rate, piloerection, pains, nausea, and many other symptoms as signs of withdrawal. Therefore, understanding the patients’ satisfaction with medications used in OUD treatment may help health care professionals make informed treatment decisions ‎[18].

Unfortunately, existing data have several limitations. Studies examining patient experiences with OUD treatment often evaluate satisfaction with the entire treatment and do not disaggregate satisfaction with the medications used [19]. When studies do measure satisfaction with the medications specifically, they are limited by the lack of scales designed for OUD treatment and that are short enough to administer regularly [20]. Qualitative studies can provide opportunities for patients with OUD to volunteer a broad range of factors shaping their satisfaction with medication [19], but they share a limitation with quantitative studies in that they often sample from those who are enrolled in treatment [12]. This approach misses the perspectives of those who cannot access treatment because of concerns over stigma or lack insurance [21]. An alternative data source is therefore needed.

### Online Health Forums

To better understand the patients’ experiences and address limitations in existing data sources, online health forums have been proven to be useful resources, as patients are not biased by the presence of a medical professional ‎[22]. Accordingly, patients seek external information sources such as health care forums or online health care communities, particularly reports of patients with similar health conditions and treatment ‎[23]. Besides, these forums also provide valuable social support, encouragement, and friendship [24,25]. In research on the efficacy of online health discussion forums for prescription drug abuse, findings imply that an online health forum is useful for assisting users with physical detoxification and opiate withdrawal [26]. Another advantage of these online platforms compared to survey data is that people decide when to post a review compared with patient satisfaction surveys. In a study on bias in patient satisfaction surveys, Dunsch et al [27] demonstrated how assessments of patient satisfaction are extremely sensitive to how the questions are framed. They also found convincing evidence of the acceptance bias, or peoples’ inclination to accept a statement regardless of its content, in particular ‎[27].

### Natural Language Processing

Analyzing data from online health forums is not without its own challenges. The text is unstructured and may contain a mix of formal and informal concepts. Moreover, across the reviews, there may be different terms used to refer to the same biomedical concepts. Natural language processing (NLP) techniques increasingly offer an alternative analytic strategy for addressing complicated interactions in large data sets, recognizing hidden patterns, and providing effective predictions in health-related texts ‎[28,29]. Several prior studies have used NLP methods to predict opioid dependency ‎[30,31], overdose ‎[32], prolonged use of opioids after surgery ‎[33], suicidality among opioid users on the online forum Reddit, or other related outcomes ‎[34]. Moreover, in analyzing health-related online review posts, Lu et al [35] discovered health-related topics using text clustering algorithms on social media data.

### Contributions

To address limitations in existing data on patient satisfaction with medications for opioid dependency, we examined online health forums. The aim of this study was to utilize NLP to detect patient satisfaction with opioid medication treatments from patient reviews in health forums that mention methadone and buprenorphine as targeted OUD medications. To the best of our knowledge, this is the first study to identify biomedical concepts influencing patients’ satisfaction with opioid medication treatments and automatically detect patient satisfaction using those concepts as model features. To achieve our goal, we utilized patient reviews from two well-known health care forums, WebMD ‎[36] and Drugs.com ‎[37], on opioid treatment medications. We also used MetaMap (an NLP and computational-linguistic tool developed by the National Library of Medicine) to extract biomedical terms used in the patients’ posts. We leveraged these terms along with the duration of treatments to train a stratified 10-fold cross-validation (CV) model to detect patient satisfaction with targeted medications.

## Methods

### Study Design

The methodology of this study consisted of four stages: (1) data collection and preprocessing, (2) identifying hidden topic models and duration of treatment, (3) identifying biomedical concepts by applying MetaMap, and (4) developing a predictive model to detect patient satisfaction with opioid medications from reviewers’ posts. We describe each of the stages in detail below.

### Data Sources

**Figure 1:**
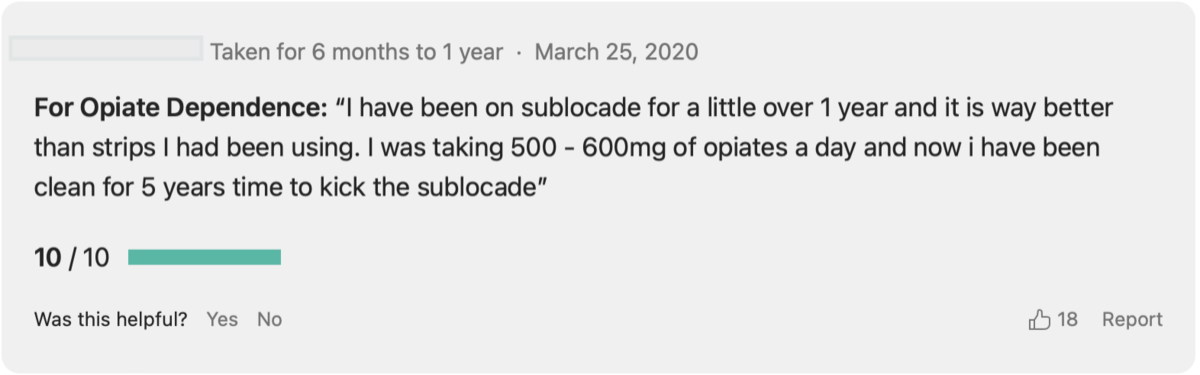
A sample review post from Drugs.com.

We used two health care forums, Drugs.com and WebMD, as our data source for this study. Both forums collect patients’ self-reported experiences for a wide range of medications. In both forums, patients can report their experiences with medication in a field called “comments.” In the WebMD forum, patients can enter their gender and age range, while the Drugs.com forum does not have an option for gender and age. In both forums, each review post includes a rating attribute for the reviewer to rate the treatment effectiveness experience as a number, which is in the range of 1-10 in Drugs.com and 1-5 in WebMD. In addition, in either forum, the reviewers can input the duration of their treatments into four categories: too short, less than 1 month, too long, and more than 10 years. WebMD also has options for collecting the “drug satisfaction” and “ease of use,” while Drugs.com does not have these two rating features. The date of reports in both forums is recorded automatically using the system. The patient’s ID is visible; however, the forums collect the patient consent to make the reported experience publicly available. Figure 1 shows a sample review post from the Drugs.com forum.

In this study, our targeted drugs were the two well-studied [38] OUD treatment medications methadone hydrochloride and buprenorphine/naloxone hydrochloride (Zubsolv, Suboxone, Subutex, and Bunavail). Methadone and naloxone (brand names: Methadose and Dolophine) are from a class of medications called opioid analgesics, whereas buprenorphine is from the partial agonist-antagonists class.

### Data Collection

We collected 4353 drug reviews from the two online forums via an automatic web scraper. We used *beautiful soup* ‎[39] in Python programming to develop the web scraper. The collected data included review posts that mentioned these two drugs (both generic and brand names for each) from 2008 to 2021, the duration of the treatment, and drug effectiveness rating. Henceforward, because the term “effectiveness” has a particular meaning in the medical literature, we refer to the numerical patient rating as “patient satisfaction.”

### Data Preprocessing

In this study, we used the whole review comment as the unit of our analysis to preserve the meaning of the patients’ review comments. We removed all posts that did not provide any comment text for the post. We then used Natural Language Toolkit ‎[40] to remove all stop words, punctuation, and non-ASCII characters. Subsequently, the words were stemmed and lemmatized for applying the topic modeling approach. This data-cleaning process improves the performance of topic modeling as it avoids repeated versions of a word in a topic and improves the detection rate significantly ‎[41]. Finally, we rescaled patients’ ratings for the two medication treatments (methadone and buprenorphine/naloxone) from the two forums for further developing the predictive model process. As stated earlier, in both forums, each review post includes a rating attribute for rating the treatment medication satisfaction by the reviewer. Drugs.com’s rating ranges from 1 to 10, whereas this rating is from 1 to 5 on WebMD. To make the rating uniform, we employed the approach proposed by Dawes [42] to rescale 1-10 ratings on a scale from 1 to 5. In this approach, 1 remains as 1 and 10 is rescaled to 5, and then the midpoint of 5.5 on a 10-point scale is changed to be 3, the midpoint of 1 is changed to 5, and so on. We then rescaled the satisfaction ratings from a 5-score scale into a binary score, in which a score of 1, 2, or 3=unsatisfied and a score of 4 or 5=satisfied [43].

### Sample Size Calculation

To identify the best features from users’ posts, we needed to determine the best random sample for the collected review posts. We first identified the ideal sample size using the finite population correction factor when sampling without replacement [44]. The formula used for calculating the sample size *n* with the limited population factor formula in statistics is as follows:

*n*=*n*_0_*N*/*n*_0_+(*N*–1)

where *N* is the population size and *n*_0_ is the size of the sample before the finite population factors are applied. We calculated *n*_0_ with the following formula:

*n*_0_=*z*^2^*p*(1–*p*)/*e*^2^

where *e* is the sampling error, *p* is the population standard deviation, and *z* is the confidence level. We then calculated the ideal size *n* for a sample with *N*=4353, *e*=0.007, *p*=0.15, and *z*=1.96 (95% confidence), yielding *n*=100. To find the best random sample of size 100, we used a stratified sample method. This approach divides the population into groups, and a proportionate number is randomly sampled from each group [44].

### Content Analysis of Drug Reviews and NLP Tools for Feature Extraction

After determining the sample size, we reviewed 100 posts via the stratified sample method manually to get a better sense of the posts’ content to determine the most suitable techniques for feature extraction. Our manual content analysis showed that the patients use both colloquial and formal medical language to express medical concepts presenting symptoms, adverse drug effects, drug effectiveness, and some social concepts (eg, social isolation or financial stress) with medication. Therefore, to identify the medical concepts, we used MetaMap to extract the medical and social concepts, particularly for formal expressions (see the Biomedical Concepts Extracted by MetaMap section below for more details). In addition, to identify the major themes in each drug review, we used the topic modeling approach. We also used vectorized text and n-grams as the baseline feature set. Furthermore, we conducted feature importance in Python to determine the contribution of each feature set to the model performance. The following sections provide more detail on each feature set.

### Biomedical Concepts Extracted by MetaMap as Features

To identify the biomedical concepts such as symptoms, drugs, and illnesses mentioned in the review posts, we employed MetaMap, a publicly available program based on NLP and computational-linguistic methods developed by the National Library of Medicine. MetaMap is commonly used in information extraction, classification, biomedical and clinical literature analysis in natural language, and unified medical language system (UMLS) concept-based indexing and retrieval. MetaMap maps biomedical text to concepts in the UMLS Metathesaurus ‎[45], which assists in organizing different vocabularies used to refer to the same biomedical concept. It takes the text and breaks it down into components that include terms, phrases, linguistic elements, and tokens through a series of modules. In a comprehensive study on MetaMap features, Aronson et al [46] reported that MetaMap has an extension of the NegEx algorithm ‎[47] to detect negated concepts.

The number of biomedical concepts extracted by MetaMap for all collected reviews was 556. To improve the performance of machine-learning algorithms, sparse features and features with low frequency for an identified concept were removed. Thus, we primarily focused on concepts with higher frequency, leaving 424 biomedical concepts on three groups of symptoms, drugs, and illnesses. The detailed procedure, including MetaMap methods and the associated results summary, is provided in Multimedia Appendix 1.

### Topic Models as Features

Topic modeling is a statistical technique that groups the words of a collection of documents based on their frequency of co-occurrence. Topic modeling’s core assumption is that a document contains a mixture of themes. To identify the main underlying themes or “hidden topics” among the patients’ posts, we utilized latent Dirichlet allocation (LDA) ‎[48], one of the popular topic modeling algorithms in NLP. LDA is a three-level hierarchical Bayesian model that models each item of a collection as a finite mixture over an underlying list of topics. The main advantage of LDA is that it is a probabilistic model with an interpretable subject and different parameters ‎[49]. Additionally, studies on online health forums dealing with breast cancer ‎[50] and Chinese social media ‎[51] revealed that LDA can be used as a feature for developing predictive models to detect postings that contain informational and emotional support automatically. However, the basic disadvantage of LDA is that it lacks objective metrics to justify hyperparameter selection ‎[52].

For our analysis, we used the LDA algorithm implemented in Python’s Gensim package ‎[53]. We utilized the Mallet function from the Gensim package. Selecting the best number of topics is important to create a meaningful set of topics. Steyvers and Griffiths [54] observed that the best number of topics varies from task to task and needs to result in the best generalization performance. Their research concluded that picking too few topics causes a vast topic, limiting the ability to discriminate. In contrast, too many topics results in topics that tend to catch unusual word combinations ‎[54]. To that extent, to determine the best number of topics and words per topic, we experimented with different numbers of topics (5, 10, 15, 20, 25, 30, and 35) and words (5, 10, 15, and 20) alongside manual tuning of the LDA parameters. We also assessed the coherence score ‎[55] corresponding to each extracted topic model calculated by Mallet and confirmed their reasonableness by manual inspection. By this method, the most meaningful set included 20 topics with 10 words per topic.

### Duration of Treatments

Fishbain et al [56] found that between 3.3% and 14.5% of long-term prescription opioid users developed an opioid dependency after an average of 22.1 months of exposure ‎[57]. Therefore, we considered the duration of opioid treatment medication as one of the predictive model’s features. On both online platforms, the users have seven choices to enter the time on the medication: less than 1 month, 1-6 months, 6 months to 1 year, 1-2 years, 2-5 years, 5-10 years, and 10 or more years.

Approximately 10% of the collected data had missing information about the treatment duration. To handle the missing data, we utilized maximum-likelihood estimation, a statistical strategy for estimating missing data based on the available data that have been seen ‎[58].

As a final step, the features extracted using NLP techniques (Table 1) were combined with the duration of treatment for developing machine-learning models to predict patient satisfaction with the two targeted opioid treatment medications: methadone and buprenorphine/naloxone (see the Data Source section for details).

**Table 1:** Four different feature sets used for the predictive model.

Input features	Baseline feature set	Feature set 1	Feature set 2	Feature set 3
Vectorized text, unigrams, and bigrams	✓	✓	✓	✓
Biomedical concepts extracted by MetaMap		✓		✓
Topic models			✓	✓
Duration of treatment			✓	✓

### Machine-Learning Algorithms

In this study, we selected six machine-learning algorithms to predict the patients’ satisfaction with OUD treatments. We chose six predictive approaches based on prior studies that have frequently produced the best prediction outcomes in classification talk ‎[59,60]. These approaches are logistic regression, the elastic network model (Elastic Net), least absolute shrinkage and selection operator (LASSO) regression, ridge regression model (Ridge), and two decision tree models, random forest and extreme gradient boosting (XGBoost).

We used stratified k-fold CV, which automatically selects training and test sets for each iteration, to train and test the machine-learning models; none of the models tune hyperparameters shared across different iterations. The k-fold CV splits the data set into k folds randomly each time and uses one dedicated fold for the test set and the rest of the folds for the training set [61]. In a stratified k-fold CV, the folds are stratified to ensure that each fold of the data set has the same proportion of observations with a given label, particularly in the case of an imbalanced labeled data set [62]. Han et al [63] recommended stratified 10-fold CV owing to its low bias and variance for assessing the performance of machine-learning algorithms. Therefore, we used a stratified 10-fold CV to train and test the models, and the average of the folds was taken to compare the metrics.

The above algorithms were fed with four novel combinations of input features (Table 1) as follows: vectorized text, which includes unigrams and bigrams (baseline feature set); vectorized text along with features from MetaMap (feature set 1); vectorized text along with features from topic models and duration of treatment (feature set 2); and lastly, vectorized text along with features from both MetaMap and topic models and duration of treatment (feature set 3). In the next section, we describe the details of the model features. We evaluated each model’s performance using general metrics of accuracy, precision, recall, F-score, and area under the curve (AUC).

## Results

### Statistical Analysis

**Figure 2:**
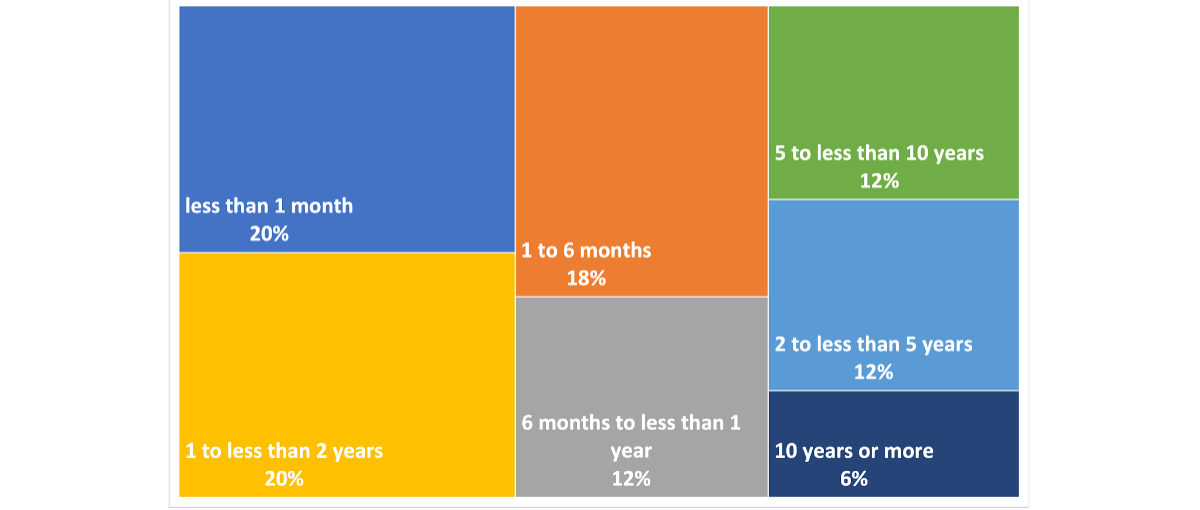
Distribution of the duration of treatment reported by patients.

Removing the posts with empty comment texts reduced the sample of 4353 posts to 4048. The average number of words per post review was 93. Among all review posts, three quarters of reviewers utilized buprenorphine/naloxone (Suboxone) and the rest used methadone. Figure 2 demonstrates the distribution of treatment duration reported by patients among six categories of time on the medication.

**Figure 3:**
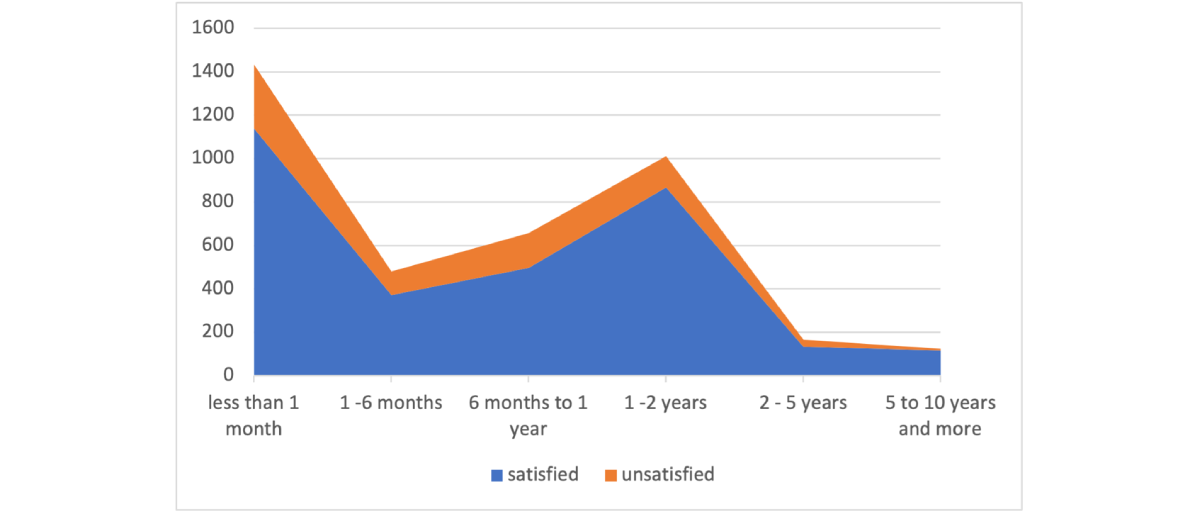
Distribution of "satisfied" and "unsatisfied" patients according to the duration of treatment.

Our statistical analysis revealed that 18% of the patients were unsatisfied with the treatment medication and 82% reported satisfaction with targeted medications. As shown in Figure 3, 36% of satisfied patients with the medications reported using the medication for less than 1 month.

After applying MetaMap to all review posts, we found that the patients mentioned symptoms such as breathing problems, dehydration, vomit, and confusion. The reviewers also mentioned illnesses such as adrenal crisis, delirium, and chronic headaches. The top 10 symptoms, other drug names, and illnesses extracted by MetaMap are summarized in Table 2.

**Table 2:** Top 10 biomedical concepts for symptoms, other drugs, and illnesses extracted by MetaMap.

Category	Biomedical concepts
Symptoms	Suffocate, breathing problem, dehydration, vomit, confusion, restlessness, disoriented, muscle weakness, mood changes, depressing
Other mentioned drugs	hydroxyzine (anxiety, nausea), luvox (OCD^a^), temazepam (insomnia), vitamins (body needs), miralax (constipation), fioricet (pain and fever reliever), Narcan (overdose), magnesium citrate (bowel movement), Adderall (ADHD^b^), Ambien (insomnia in adults)
Illness	adrenal crisis, delirium, reflex sympathetic dystrophy, cyst, hydrocephalus, chronic headaches, sciatica, fibromyalgia, herniated discs, degenerative joint disease

^a^OCD: obsessive compulsive disorder.

^b^ADHD: attention deficit and hyperactivity disorder.

As depicted in Table 2, reviewers mentioned other drugs in their posts, such as hydroxyzine, which is used for anxiety and nausea, and temazepam, which is helpful for insomnia. After applying topic modeling methods, the top four hidden topics extracted by LDA were an oral sensation, side effects, insurance, and doctor visit. Table 3 demonstrates the top 10 words associated with each cluster of topics.

**Table 3:** Four meaningful topics and associated words extracted by Mallet.

Topic	Top 10 associated words
Oral sensation	dissolve, experience, tongue, back, minute, mouth, cheek, form, considerable, night
Side effects	bad, anxiety, depression, sober, luck, meditation, write, panic, person, properly
Insurance	insurance, find, cover, anymore, strong, worry, company, doctor, couple, list
Doctor visit	doctor, thing, put, prescribe, amazing, blood, sublingual, leave, worried, shot

### Prediction Model Performance

Table 4 shows the predictive model performance through four different feature sets: vectorized text, including unigrams and bigrams (baseline feature set); vectorized text along with biomedical concepts (feature set 1); vectorized text along with topic models and duration of treatment (feature set 2); and lastly, vectorized text along with biomedical concepts, topic models, and duration of treatment (feature set 3). Our feature importance analysis revealed that text alone had higher importance in the models’ performance than features extracted by MetaMap, topic models, and the duration of treatments. After feeding each model with a different set of features, we found a slight improvement in the F-scores of the predictive models compared to the baseline model, except for the Ridge classifier with a small deterioration.

**Table 4:** Performance (F1-scores) of six classifiers for each combination of features.

Model	Baseline feature set	Feature set 1	Feature set 2	Feature set 3
Logistic regression	90.2	90.3	90.2	90.5
Elastic Net	90.2	90.2	90.2	90.6
LASSO^a^	90.3	90.1	90.3	90.5
Random forest	90.0	90.0	90.0	90.0
Ridge classifier	90.8	90.2	90.7	90.6
XGBoost^b^	89.9	90.2	90.1	90.2

^a^LASSO: least absolute shrinkage and selection operator.

^b^XGBoost: extreme gradient boosting.

**Figure 4:**
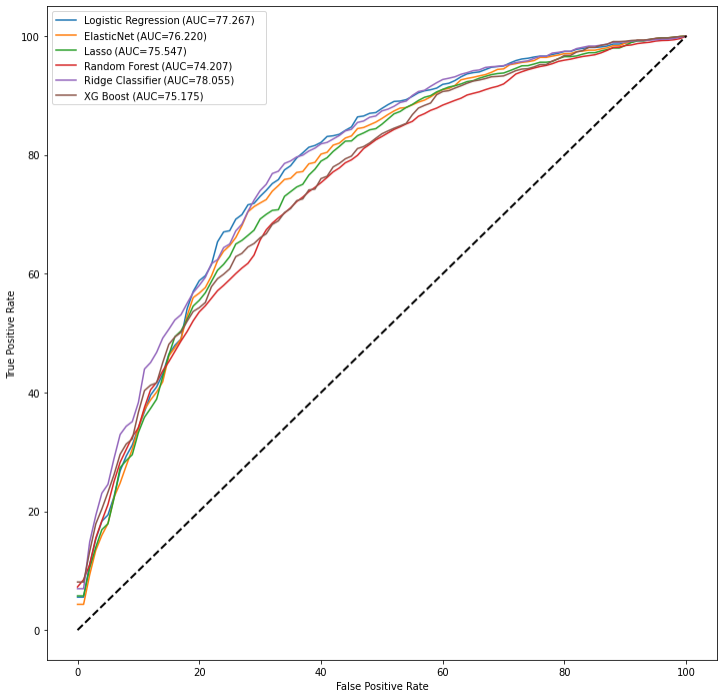
Receiver operating characteristic plots showing the performance comparison among six classifiers, including feature set 3. AUC: area under the curve; LASSO: least absolute shrinkage and selection operator; XGBoost: extreme gradient boosting.

The receiver operating characteristic curves in Figure 4 compare different classifiers for different feature sets. Comparing the AUC values shows that the logistic regression model (AUC=78.8) outperformed the other models. The complete curves for all classifiers and feature sets are available in Multimedia Appendix 2.

**Figure 5:**
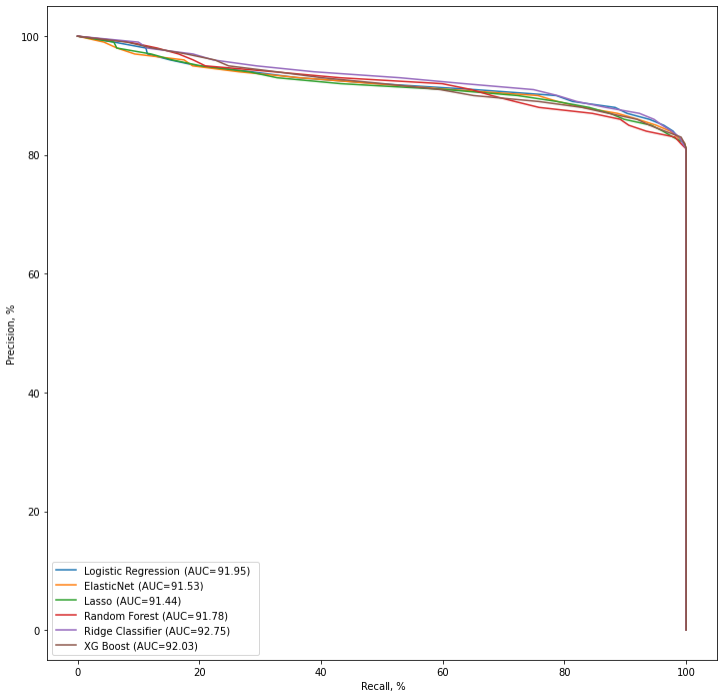
Comparison of the precision-recall curves of all models, including feature set 3. AUC: area under the curve; LASSO: least absolute shrinkage and selection operator; XGBoost: extreme gradient boosting.

Figure 5 shows the precision-recall curves of all models on the different feature sets. The AUC values were calculated based on the average of the AUC of each curve. Comparing the prediction scores shows that all models had a close range of scores. As shown in Table 4, the F-score for all models ranged from 89.9% to 90.8% for the baseline feature set, biomedical concepts, topic models, and duration of treatment feature combination set. The Ridge classifier model scores, in general, were better than the other models’ scores. Adding biomedical concepts, topic models, and duration of treatment as features individually and in combination improved the performance measures of the logistic regression, Elastic Net, LASSO, and XGBoost models, whereas there were no changes for the random forest model. The results also revealed that the Ridge classifier gained the highest performance (F-score=90.8) by having the baseline feature set as its input.

## Discussion

### Principal Findings

In this study, we collected review posts from two online health-related forums, Drugs.com and WebMD, to investigate creating a predictive model to automatically identify patient satisfaction with OUD treatment medications. The data source presents challenges for analysis because it is unstructured, patient-generated text. We demonstrated how analysts can use MetaMap to detect biomedical concepts alongside an NLP rule-based algorithm and topic modeling (unsupervised NLP algorithm) to detect patient satisfaction. Our analysis of feature importance uncovered that the text alone as a baseline feature is a significant input variable to predict the output variable. This is aligned with the results coming from integrating biomedical concepts extracted from MetaMap and topic models with other features such as duration of treatment, which led to adding slight value to the predictive model performance. Our study also found that compared to other models, Elastic Net, a regularized regression method, improved the most upon the addition of biomedical features along with other features, which is in line with Marafino et al’s [64] study on biomedical text classification on nurses’ notes. The F-score ranged from 89.9 for the XGBoost model to 90.8 for the Ridge classifier model, including the baseline feature set. The AUC value ranged from 74.0 (random forest) to 78.8 (logistic regression). When training models with different machine-learning models, we manually considered some alternative values for the models’ parameters, but this resulted in no significant improvements. For instance, we manually adjusted the number of iterations to the logistic regression, Elastic Net, and LASSO models from 1000 to 50,000, but we noticed no significant change.

To the best of our knowledge, this study is the first to identify the biomedical concepts from reviews of opioid medication treatments among patients who have been struggling with the issue of OUD treatment and to predict patient satisfaction with these medications. This is critical given previous research showing the importance of medications for shaping experiences with opioid dependency treatment [11].

This study aligns with the findings from other research, while also underscoring the added value of analyzing reviews from online health forums. Our study showed that patients who used different forms of buprenorphine/naloxone (Suboxone) and methadone for their OUD mentioned numerous symptoms, which is in line with the findings of Perlogizzi et al [65] on opioid withdrawal symptoms, who showed that these symptoms are both a motivator for continuing opioid usage and a barrier to stopping them [65]. Symptoms may also reflect side effects, which are commonly ascertained in self-reported surveys of medication satisfaction and volunteered during interviews and focus groups [12,20]. Oral sensation when ingesting the medication and frequency of doctors’ visits also appear regularly in patient reports of their experiences [12]. Notably, insurance was revealed as a topic appearing in the reviews, and words related to this topic included the term “worry,” possibly indicating concerns about having insurance to assist with financial barriers to treatment. Approximately one-fifth of those who experience opioid dependency lack health insurance coverage, which increases their risk of forgoing treatment [20]. Insurance coverage and concerns about other financial barriers are rarely considered in medication satisfaction scales, however, which highlights the contributions of monitoring online health forums to capture patient satisfaction more fully.

### Limitations

This study has several limitations that may impact the results. The reviews we incorporated may not reflect the viewpoints of the population of patients with OUD fully because we only collected reviews from two websites (WebMD and Drugs.com) and we cannot determine the demographic or medical background of the reviewers. Moreover, because we were limited in only using the formal names of medications as keywords, we may have missed more colloquial discourse that refers to these using slang. Other platforms such as Twitter may provide sufficient information to infer background characteristics and capture more colloquial references to the medications, but previous work found that such data were not as well-structured or relevant as review text [43].

By incorporating reviews from two different websites, this imposed restrictions in how we structured and processed the data. Because patient satisfaction was measured on one site on a 5-point scale and measured on a 10-point scale in the second site, we had to rescale the ratings to make them uniform (on a scale from 1 to 5), which resulted in the loss of some information. Besides the text of the review, we also only had one feature present in both websites (duration of treatment) to use as one of the predictive model’s features. To further assist in managing text from two different websites that may include a mix of biomedical and informal language, we used a combination of NLP techniques. We used MetaMap, which is useful for identifying biomedical concepts because it leverages the UML Metathesaurus, but it may still fail in recognizing and mapping a disease name effectively [66]. We also used topic modeling but used it on the entire review, and each review may contain different sentences with different sentiments and topics, as people reflect on their lives before or during treatment. Despite these limitations, we achieved high accuracy, and the resulting algorithm may still help address a complex crisis entangled with public health as well as with social and economic welfare, especially in the treatment of pain, a major health issue [67].

### Future Work

Based on the current methods of this study and the limitations mentioned above, several future directions are suggested to build on this research. Foremost, adding demographics such as the gender of the reviewer and evaluating whether these interact with treatment can play an essential role in testing whether there are demographic disparities in responses to opioid treatments. Furthermore, future work could extend the analysis to explore the relationship between online opioid treatment reviews by patients and clinical notes by health care providers. In addition, sentiment analysis could be performed and added as a feature in addition to hidden topics. Moreover, applying advanced filtering techniques to the reviews may improve retrieving text more relevant to the subject of study and refining contextual polarity to better grasp what a word or phrase implies in a given context. Lastly, word embeddings and deep-learning methods are other suggestions for future work to investigate the improvement in the model’s performance.

### Conclusions

To address the need to more fully capture patients’ experiences with medications for OUD treatments, this study used different models and classifiers to predict patient satisfaction using reviews from two online health forums. As a part of this research, we performed topic modeling and found that patients’ main concerns regarding OUD treatments are insurance, anxiety/depression, doctor visits, and types of medications. Insurance is a topic rarely covered in scales to measure medication satisfaction during OUD treatments, despite one-fifth of those with OUD lacking health insurance. We also found that including treatment duration, hidden topics, and biomedical concepts such as symptoms, drug names, and illnesses was beneficial in developing some of the predictive models, specifically the Elastic Net model, for this study. Despite the data source comprising unstructured patient-generated text, these methods showed that we could analyze patient reviews and predict patient satisfaction with an opioid dependency with an F-score of approximately 90%. This result offers a promising method for automatically extracting information from patients’ comments on health care web forums.

## Appendix

Multimedia Appendix 1 MetaMap methods and results of its application to the data of this study.

Multimedia Appendix 2 Area under the receiver operating characteristic (ROC) curve (AUC) results for different classifiers with four feature sets.

## Abbreviations

AUC: area under the curve
CDC: Center for Disease Control
CV: cross-validation
LASSO: least absolute shrinkage and selection operator
LDA: latent Dirichlet allocation
MAT: medication-assisted treatment
NLP: natural language processing
OUD: opioid use disorder
UMLS: Unified Medical Language System
XGBoost: extreme gradient boosting
