# Supplementary material for: Predicting Patient Satisfaction With Medications for Treating Opioid Use Disorder: Case Study Applying Natural Language Processing to Reviews of Methadone and Buprenorphine/Naloxone on Health-Related Social Media
Source: JMIR Infodemiology. 2023 Jan 23;3:e37207. doi: 10.2196/37207 (PMC9987197; doi:10.2196/37207)
Supplement: Multimedia Appendix 1 [file infodemiology_v3i1e37207_app1.pdf]

Appendix 1

This appendix includes two parts. The first part contains how to use MetaMap and the other part encloses the results of applying MetaMap in this study for the interested reader.

What is MetaMap?

MetaMap maps (matches) text (from papers, queries) into the Metathesaurus UMLS concepts. The text is taken through a set of modules and broken down into components that include words, sentences, lexical elements, and tokens. Variants are produced from the resulting phrases, and candidate concepts from the UMLS Metathesaurus are retrieved and assessed against their phrases. The resulting concepts are arranged, known as a final mapping, in such a way as to cover the text better.
MetaMap is entirely customizable, and option flags control its behavior.

Different methods can use MateMap:

- Use MetaMap interactively
- Use Batch MetaMap
- Use MetaMap via Web API
- Run MetaMap locally

Interactive MetaMap: The user uses a web-based interface to enter text, set up parameters, and get the results.

Batch MetaMap: In this method, a large data set can be processed by uploading the input file and selecting options relevant to the individual programs. The process will be scheduled to be done. After completing the process, the user will be notified via email with instructions on downloading the results.

Web API: The user can use a Java-based API instead of a web-based interface. Like the batch metamap, the API submits the job to the Scheduler Batch facilities. Also, sending the job to an Interactive facility for processing is possible. The latest version of the API is *SKR_Web_API_V2_4.jar*

Local MetaMap: Nih.org has provided tools to install and run MetaMap locally. In this case, using the website to process the text is optional.

Downloading and installation of MetaMap and Java API

For using MetaMap locally, two sets of programs should be downloaded and installed:

(1) MetaMap Service: Known as MetaMap, it is a complete set of programs and dictionaries required to process the text.

(2) MetaMap Java API: This API uses the MetaMap service to request text processing. Before using this API, the MetaMap service should be installed and running.

Because most parts of the two above programs are written in Java, upgrading Java Runtime Environment to the latest version is recommended. (https://www.java.com/en/download/)

The most recent versions of MetaMap and the API as of working on this research are shown in the table:

Operating System	MetaMap	MetaMap Java API
Unix	(2020) https://metamap.nlm.nih.gov/download/public_mm_linux_main_2020.tar.bz2	(2020) https://metamap.nlm.nih.gov/download/public_mm_linux_javaapi_2020.tar.bz2
Windows	(2014) https://metamap.nlm.nih.gov/download/public_mm_win32_main_2014.zip	(2014) https://metamap.nlm.nih.gov/download/public_mm_win32_javaapi_2014.zip
Mac OS	(2018) https://metamap.nlm.nih.gov/download/public_mm_darwin_main_2018.tar.bz2	(2016v2) https://metamap.nlm.nih.gov/download/public_mm_darwin_javaapi_2016v2.tar.bz2

Installation on Unix or macOS machines:

For Unix and macOS operating systems, use the terminal and make the d directory a root directory for your MetaMap files. You should obtain a username and password and verify it to use some sources of MetaMap, including the MetaMap program and MetaMap Java API. After logging in to the system, you can download the required files from the above links.

MetaMap installation:

After downloading both MetaMAP and Java API files, follow these steps:

- Move the downloaded files to the root directory of MataMap
- Unzip the downloaded file with this command:
  bunzip2 -c public_mm_linux_main_2020.tar.bz2 | tar xvf -
  For macOS machines, use this command instead:
  bunzip2 -c public_mm_darwin_main_2018.tar.bz2 | tar xvf -

- This command unzips the file and makes a directory under the name public_mm. Use the command: cd public_mm

To change the directory.

- Use the following command to install the package: *./bin/install.sh*
- During the installation, you will get several messages regarding the installation steps. In the end, the last message will be: Install complete.

Running MetaMap:
After the successful installation of MetaMap, you can run the MetaMap service.
Starting the MetaMap Server:
Two commands can be used to start the service:
./bin/skrmedpostctl start

./bin/wsdserverctl start

The first command starts the SKR/Medpost Part-of-Speech Tagger Server, while the second command starts an optional Word Sense Disambiguation (WSD) Server.

Stopping the MetaMap Server:
Two commands similar to start commands can be used to stop the service:
./bin/skrmedpostctl stop

./bin/wsdserverctl stop

To verify the services that are currently running, one can use the command: *ps -if | grep Java*

You can verify if the server is working by using a similar command.
Echo "lung cancer" | *./bin/metamap -I*
You may open a new terminal window or tab to issue that command.

MetaMap Java API installation:

Similar to the MetaMap, the downloaded file for the Java API should be extracted from the same folder of MetaMap:
bunzip2 -c public_mm_linux_javaapi_2020.tar.bz2 | tar xvf -
For Linux machines and
bunzip2 -c public_mm_darwin_javaapi_2016v2.tar.bz2 | tar xvf -
For macOS computers, use this command: *cd public_mm*
To change the directory, use this command: *./bin/install.sh*

Running the MetaMap Java API:

After installing both programs, you can use the Java API to analyze a text. Before running the API, make sure that the MetaMap Server is running.
The general command for running MetaMap is:
./bin/metamap [options] InputFile OutputFile

MataMap reads the input file and analyzes it according to the options specified. If the output file is not specified, it creates an output file by appending ".out" extension to the end of the input file.

Some MetaMap sample results:

Meta Mapping (790):

790 METHADONE (Methadone) [Organic Chemical, Pharmacologic Substance]

Phrase: for 4yrs I thought opiates

Meta Mapping (775):

645 I- (Iodides) [Inorganic Chemical]

645 Thought (Thinking, function) [Mental Process]

812 Opiates (Opiate Alkaloids) [Hazardous or Poisonous Substance, Organic Chemical,

Pharmacologic Substance]

Meta Mapping (775):

645 I- (Iodides) [Inorganic Chemical]

645 Thought (Thinking, function) [Mental Process]

812 Opiates, NOS (Opiate Assay) [Laboratory Procedure]

Meta Mapping (775):

645 I- (Iodides) [Inorganic Chemical]

645 Thought (Thinking, function) [Mental Process]

812 Opiates [Hazardous or Poisonous Substance, Organic Chemical, Pharmacologic

Substance]

Meta Mapping (775):

645 I- (Iodides) [Inorganic Chemical]

645 Thought [Idea or Concept]

812 Opiates (Opiate Alkaloids) [Hazardous or Poisonous Substance, Organic Chemical, Pharmacologic Substance]

Meta Mapping (775):

645 I- (Iodides) [Inorganic Chemical]

645 Thought [Idea or Concept]

812 Opiates, NOS (Opiate Assay) [Laboratory Procedure]

Meta Mapping (775):

645 I- (Iodides) [Inorganic Chemical]

645 Thought [Idea or Concept]

812 Opiates [Hazardous or Poisonous Substance, Organic Chemical, Pharmacologic Substance]

Meta Mapping (775):

645 I NOS (Blood group antibody I) [Amino Acid, Peptide, or Protein, Immunologic Factor]

645 Thought (Thinking, function) [Mental Process]

812 Opiates (Opiate Alkaloids) [Hazardous or Poisonous Substance, Organic Chemical, Pharmacologic Substance]

Meta Mapping (775):

645 I NOS (Blood group antibody I) [Amino Acid, Peptide, or Protein, Immunologic Factor]

645 Thought (Thinking, function) [Mental Process]

812 Opiates, NOS (Opiate Assay) [Laboratory Procedure]

Meta Mapping (775):

645 I NOS (Blood group antibody I) [Amino Acid, Peptide, or Protein,Immunologic Factor]

645 Thought (Thinking, function) [Mental Process]

812 Opiates [Hazardous or Poisonous Substance,Organic Chemical,Pharmacologic Substance]

Meta Mapping (775):

645 I NOS (Blood group antibody I) [Amino Acid, Peptide, or Protein, Immunologic Factor]

645 Thought [Idea or Concept]

812 Opiates (Opiate Alkaloids) [Hazardous or Poisonous Substance, Organic Chemical, Pharmacologic Substance]

Meta Mapping (775):

645 I NOS (Blood group antibody I) [Amino Acid, Peptide, or Protein,Immunologic Factor]

645 Thought [Idea or Concept]

812 Opiates, NOS (Opiate Assay) [Laboratory Procedure]

Meta Mapping (775):

645 I NOS (Blood group antibody I) [Amino Acid, Peptide, or Protein,Immunologic Factor]

645 Thought [Idea or Concept]

812 Opiates [Hazardous or Poisonous Substance, Organic Chemical, Pharmacologic Substance]

Phrase: was

Phrase: bad,

Meta Mapping (1000):

1000 Bad [Qualitative Concept]

Meta Mapping (1000):

1000 BAD (Brachial Amyotrophic Diplegia) [Disease or Syndrome]

Phrase: this methadone

Meta Mapping (1000):

1000 METHADONE (Methadone) [Organic Chemical, Pharmacologic Substance]

A complete list of MetaMap results for this study:

Below is a complete list of the extracted 556 biomedical concepts by applying MetaMap in our study.

**Sign or Symptoms (Sosy)**	**Disease or Syndrome (dysn)**	**Drug names**
Chronic Pain	MEDS	Heroin
Constipated	FIBROMYALGIA	OPIOIDS
PAIN	chronic pain hip	Hydrocodon
Chronic back pain	PERIPHERAL NEUROPATHY	ASCEND
Breakthrough Pain	Relapse	Opiate
Fatigued	Secondary adrenal insufficiency	MORPHINE
Exhausted	Pituitary	FENTANYL
heart problems	OSTEOPOROSIS	Adderall
EXHAUSTION	Staring	Ambien
SYMPTOMS	MIGRAINE	CLOT
SPASMS	HYPOGONADISM	Percocet

Deep pain	allergic rash	Dilaudid
pain night	Normal Tolerance	Neurontin
painful hip	SCOLIOSIS	Suboxone
Withdrawal Symptoms	restless leg	Lortab
PAIN JOINT	Infarction, Myocardial	Opioid
Radicular pain	Degenerated disc disease	OxyContin
JOINT PAIN	NEUROPATHY, SCIATIC	OXYCODONE
Tired	KIDNEY STONE	Pain medicine
pain heart	Migraine Headaches	Norco
pain spine	degenerative disc	dope
BONE PAIN	NEUROPATHY	Benzodiazepine
Cancer Pain	ARTHRITIS	Subutex
Sick	HEART ATTACK	Steroid
lips numb	REFLEX SYMPATHETIC DYSTROPHY	Opiates
pain breathing	RSD - Reflex sympathetic dystrophy	Sugar
CONSTIPATION	temporal pain	Vicodin
DORSAL BACK PAIN	CIRRHOSIS	Never Pain
KNEE PAIN	Cavities	Dilaudid Pill
Painful	staggered	DOCUSATE SODIUM

weight problem	COMA	percocets
Catch	tooth cavity	Roxicodone
NERVE PAIN	Abscesses	TESTOSTERONE
DIARRHOEA	Spinal Arthritis	Pain killers
SLEEPLESSNESS	MULTIPLE SCLEROSIS	Klonopin
BACK PAIN, SEVERE	DEGENERATIVE JOINT DISEASE	Opana
right leg pain	Lupus	Lorcet
cold sweats	wasted	Opana ER
Restlessness	chronic leg ulcers	Pain-Free
AGITATION	Herniated Disk	Tylenol
life quality	Blood Pressure, High	Meth
Oral Pain	drug withdrawals	pain Drugs
BACK PAIN	DEHYDRATION	Painkillers
tired time	SEIZURE GRAND MAL	OPIUM
back severe pain	disc degenerative disease	Metamucil
BACKPAIN	MIGRAINES	Breath-Away
Lethargic	multiple condition	Methadose
Headaches	Phantom pain	Marihuana
constipate	ARACHNOIDITIS	Xanax
Night pain	chronic lower back pain	Tramadol Pill

Moderate pain	OSTEOARTHRITIS	ALCOHOL
Trouble sleeping	Degenerative disc disease	Vitamin
Trouble breathing	CYST	Benzodiazepines
side pain	Herniated Disks	Cancer Drug
injections pain	Flashes	Agent Orange
restless	CRPS	barley
feet sore	Disease	GABAPENTIN
Shakes	INTERSTITIAL CYSTITIS	Oily
Dizzyness	neuropathies	MAGNESIUM CITRATE
tooth ache	BENDS	COCAINE
pain lower leg	HAIR LOSS	Mobic
VOMITING	headache migraines	Vicodin Pill
pains	Neuralgia, Trigeminal	Pain Relievers
SCIATICA	COLITIS	CODEINE
PAIN HEAD	Chronic headaches	Kadian
KIDNEY PAIN	Arrythmia	Advil
kidney problems	Herniated Discs	Fioricet
sick stomach	DRUG WITHDRAWAL	Crack Cocaine
LETHARGY	Outlet Syndrome, Thoracic	Ultram
Liver pain	Carpal tunnel	HYDROMORPHONE
DIZZY	Sleep apnoea	Miralax

lower backpain	Lumbar Degenerative disc disease	Valium
Aches	SCLERODERMA	Contin, MS
NIGHTMARES	Degenerative Disease	LEVORPHANOL
ill	Itching rash	pain reliever
Weak	Cold	Compazine
MUSCLE SPASM	hiv-infection/aids	Balance
Nightmare	chronic Lumbar pain	Morphine sulphate
pain liver	NEUROPATHY, PAINFUL	Oxycontin Pill
Long-lasting pain	spine disease	Duragesic
MOUTH Itching	SPONDYLOSIS	Fibre
BODY PAIN	Chronic Joint disorder	Sleeping Pills
spine pain	DISC DISEASE	Oxycodone Pill
Excruciating pain	Chronic low back pain	METHAMPHETAMINE
Withdrawal Symptom	Diabetes	Zanaflex
Seizures	Chronic spinal cord Disease	water solution
Sleep disturbances	previous Heart attack	LIDOCAINE
SWELLING	Viral Meningitis	Morphin
Intractable pain	Arthritis, Degenerative	Lortab Pill
Chronic constipation	Spondylolysis	Mirapex
Miserable	Phantom Pains	Morphine Pill

HEADACHE, SEVERE	Chronic Diseases	Pain management drugs
hangovers	WITHDRAWAL HEADACHE	Lyrica
CERVICAL PAIN	back Disease	Avid
NAUSEA	chronic headache migraine	Fiorinal
ailments	headaches stress	Pain-relief Medication
HOT FLASHES	Anorexic	BUPRENORPHINE
Feeling tired	Chronic pain syndrome	BuTrans
pain worsening	CARPAL TUNNEL SYNDROME	Zubsolv
Muscle spasms	arrested	NALOXONE
Nausea/vomiting	PNEUMOTHORAX	Vistaril
SEIZURE	muscle weakness	DOPAMINE
FOOT PAIN	appetite loss	Phentanyl
kidneys pain	Dystrophy, Muscular	Subutex Pill
CHILLS	RHEUMATOID ARTHRITIS	Afrin
TIREDNESS	Disease, Spinal	Aftera
HEADACHE	chronic condition	TRAMADOL
FACIAL PAIN	Slipped Disks	CLONIDINE
Lightheadedness	Nerve Root Disease	codeine syrup
Foot joint pain	chronic Migraine headaches	Suboxone Pill
Confusion	Syndrome	CLONAZEPAM
problem signs	Adhesive arachnoiditis	MORPH

Arthritis Pain	Rare Disease	pain child
MUSCLE PAIN	Ruptured disk	Actiq
PAIN NECK	spine condition	TEMAZEPAM
sick feeling	Curb	Talwin NX
Sickness	Chronic Head Pain	Methadone+Metabolite
FORGETFULNESS	FLU	TRAZODONE
HEAD PAIN	Headache Disease	sleeping pill
Flare	SHINGLES	Norco Pill
RASH	chronic Migraines	child pain
Cramps	chronic knee pain	IBUPROFEN
Cramps - leg	Plague	Phenergan
bad taste mouth	HEPATITIS	Alli
DISCOMFORT	Chronic Insomnia	OATMEAL
Unbearable Pain	LIVER FAILURE	MINT
FATIGUE	Rebound headache	FLAXSEED
weight Issue	Genetic Condition	PROzac
NECK PAIN	ULCER STOMACH	BEN
Swollen feet	ACUTE RENAL FAILURE	Heroin Pill
Nauseated	ANEMIA, SEVERE	LIME
Nose run	TOOTH DECAY	Narcan
Pain, Referred	Headaches, Chronic Daily	NALTREXONE

Discharge	Cervical arthritis	Coke
Chronic Lumbar pain	headache cough	Vicodin ES
vomit	UTI - Urinary tract infection	Tylenol PM
Nervous	Infection, NOS	Tylox
MEMORY LOSS	"Sick" headaches	Acetominophen
Lumbar back pain	Medication withdrawal	Steroids
COUGH	Other Disease	Roxicet Pill
MYALGIA	SJOGRENS SYNDROME	Pain medicines
STINGING	Tooth condition	Depakote
Pains, Lower Back	Kidney Stones	Vivitrol
spell	strangle	FISH OIL
PAIN BONE	GRIP	VITAMINS
nerves pain	Medicine withdrawal	Nasea
Burning feet	CAPS	Darvocet
Gas	LIVER DISEASE	NAPROXEN
Pain symptoms	Canker Sores	Ambien Pill
constipating	alcohol effect	stay awake
PAIN STOMACH	Disk Degeneration	Hydrocodone Pill
pain tumor	Disorders	meds rx
weight loss problem	TENDONITIS	rx med
problems weight	bone spur	VERT

shake	arthritis shoulders	Zomax
MUSCLE WEAKNESS	'Crack baby'	Motrin
headache severe	Mouth rot	Cymbalta
FEVER	Roaring	Topamax
sore	Vulvodynia	pain adult
Low backpain	Chronic osteoarthritis	Ativan
MOUTH PAIN	Drug Rashes	Phenobarbitol
INSOMNIA	cold symptoms	arsenal
Lumbar pain	DOORS	austin
burn pain	Struck	Morphine.free
SITTING PAIN	painful period	MIRTAZAPINE
TEMPORAL HEADACHE	Strokes	VENLAFAXINE
PAIN THROAT	Disease, Lyme	DIAMORPHINE
Spasm	lymes disease	Librium
BACK PAIN, UPPER	KNEE ARTHRITIS	Prometazin
PAIN MOUTH	PIAN	Sublocade
Dorsal Neck Pain	Severe dehydration	Xanax Pill
loss feeling	SOFT	THEBAINE
Weight symptom NOS	Facet arthritis	Alkaloid
LEG PAIN	WASTING	Benadryl
Ache	ENDOMETRIOSIS	steroid Medications

Chronic Pain symptoms	ATE	Rebalance
pain Injury	Miscarriage	Black tar
Wind NOS	BRONCHITIS, CHRONIC	Dragon
Birth pain	Heart trouble	Skag
charm	Nephropathy	alcohol substance
Flush	drugs withdrawal	DOXIL
Whole body pain	STROKE	Percocet Pill
Miserable Pain	PEPS	TOBACCO
bitter taste	COMMON COLD	Nicorette
Nauseous	curbing	MEND
Cancer-Related Pain	Tearing	Smack
Dull	ASTHMA	morphines
Fit, NOS	RESTLESS LEGS	Demerol
Feeling Cold	CROHN'S DISEASE	Hydrocone
large pupils	ENDOCARDITIS	Thrive
Confused	INFLAMMATORY BOWEL DISEASE	TAC
Stomach pain	Crohn's	popper
SHOULDER PAIN	Prolonged QT Syndrome	ACETAMINOPHEN
breathing chest pains	CIRRHOSIS LIVER	Zubsolv Pill
SNEEZING	SLEEP WALKING	Bunavail

nauseating	Sleeping Illness	Buprenophine
Drug Withdrawal Symptoms	HEADACHE, MIGRAINE	HYDROXYZINE
Upset Stomach	Chronic Disease	cramping diarrhea
Chronic vomiting	Hepatitis C	LIFE-GUARD
Gasp	Hive	PURSLANE
worse headaches	chronic Knee joint pain	Lexapro
feeling loss	irritation tongue	Luvox
AGITATED	HIVES	Probuphine
depression Pain	Extensive Disorder	Oxis
Burning	DERMATITIS	Prevail
HEARTBURN	KIDNEY DISEASE	Buprenorphine Buccal Film
headaches temples	MOTION SICKNESS	sleep tabs
physical symptoms	HYDROCEPHALUS	Dehydrated
RUNNY NOSE	OBESITY	
Back Aches	Ischaemic colitis, NOS	
winding	MEND	
Throbbing pain	Grippe	
hunger pains	rupture disc	
muscle cramps foot	IRRITABLE BOWEL SYNDROME	
HESITANCY	Sleep Talking	

NIGHT SWEATS	ADRENAL INSUFFICIENCY	
TREMOR	ADRENAL CRISIS	
suffocate	diarrhea anxiety	
WEAKNESS	heave	
rundown	flu stomach	
JOINT ACHE	Irregular heartbeat	
BACK PAIN, LOWER	Restless Legs Syndrome	
HUNGRY	itchy rash	
damage skin	Hot spots	
Tongue symptoms	physical Sickness	
Rest pain	Serotonin syndrome	
FLU-LIKE SYMPTOMS	Slipped Discs	
ABDOMINAL PAIN	bending	
Itchy foot	Early menopause	
spells		
dry heave		
Lightheaded		
catching		
opiate symptoms withdrawal		
Wasting		
Chronic pelvic pain		

Despair		
BODY ACHE		
Distress, Respiratory		
Troublesome Pain		
headaches lumbar puncture		
Swallowing problem		
extremities numbness		
NUMBNESS		
MALAISE		
heart trouble		
flush face		
Tense		
SHAKING		
breathing stop		
Muscle aches		
symptoms diarrhea		
spots		
Ached		
back chronic pain		
pelvis pain		
opiate withdrawal symptoms		

NERVOUSNESS		
FLUSHING		
cravings sugar		
back symptoms		
MUSCLE ACHE		
Hot flush		
Aching pain		
Soreness		
Suffering, Physical		
Blackout		
Acute constipation		
HAND PAIN		
Disoriented		
Back Pain symptoms		
Hive		
sore tongue		
OEDEMA		
jittery feeling		
pain penis		
Urinary hesitation		
Sore Throats		

HANGOVER		
Pains, Abdominal		
pain sit		
Frequent headaches		
Rashes		
MOUTH TENDERNESS		
sleeping difficulty		
Affective Symptoms		
Dazed		
Illness		
SNEEZE		
Depression symptoms		
Aching		
discomforts		
symptoms anxiety		
neoplasms pain		
Pains, Back		
Acute Pain		
symptoms depression		
ILL FEELING		
SLUGGISHNESS		

vomited		
Chill		
dilated pupils		
TONGUE PAIN		
Feeling sick		
sour taste		
STOMACH ACHE		
extreme fatigue		
laziness		
panic symptoms		
painful arm		
Burp		
Energy Reduction		
Withdrawal complaint		
PAIN KNEE		
swollen mouth		
TREMORS		
nose itching		
digestive problem		
deep breath		
