# Supplementary material for: Predicting Patient Satisfaction With Medications for Treating Opioid Use Disorder: Case Study Applying Natural Language Processing to Reviews of Methadone and Buprenorphine/Naloxone on Health-Related Social Media
Source: JMIR Infodemiology. 2023 Jan 23;3:e37207. doi: 10.2196/37207 (PMC9987197; doi:10.2196/37207)
Supplement: Multimedia Appendix 2 [file infodemiology_v3i1e37207_app2.pdf]

Appendix 2

The figures below show different classifiers ROC-AUC results with four feature sets.

(1) Baseline feature set: Text without any extra features

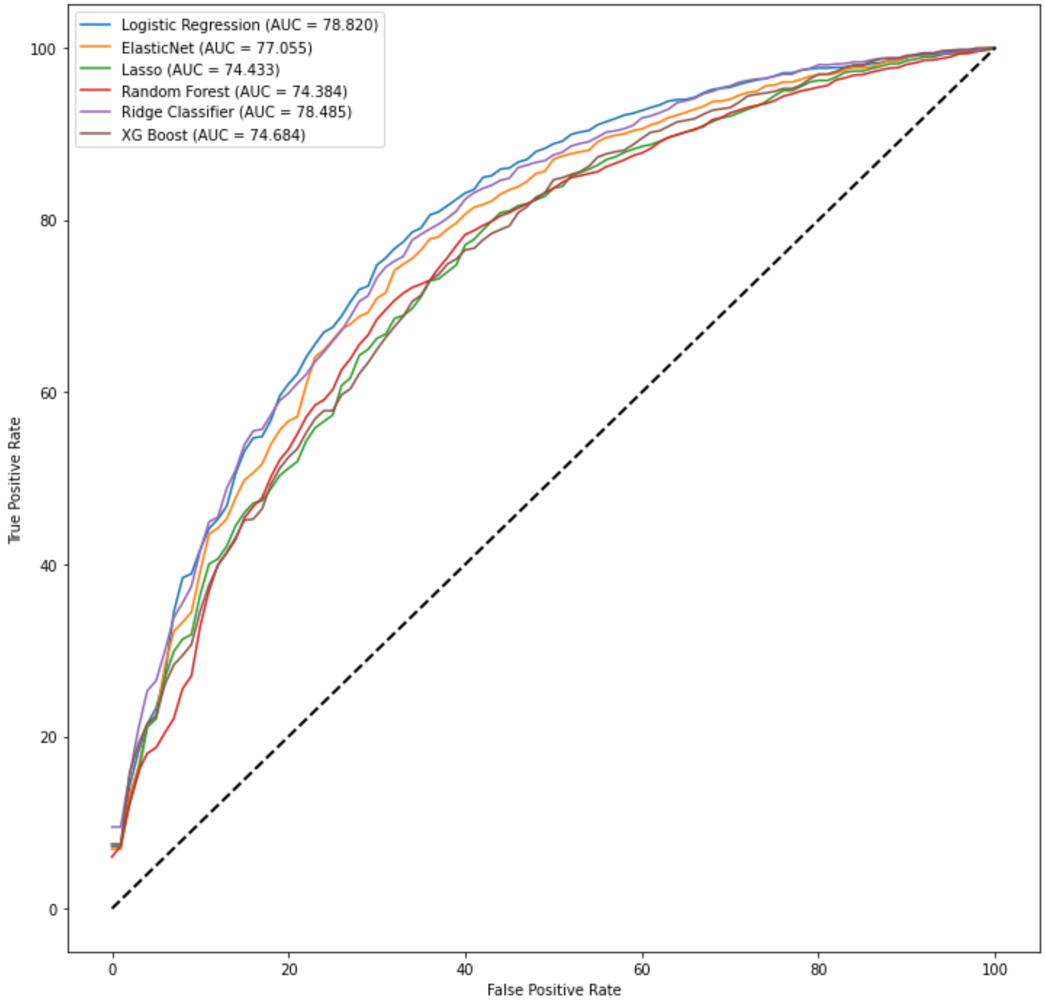

Figure 1. ROC plot shows the performance comparison among six classifiers including baseline feature set. Blue curve: Logistic Regression (AUC =78.820), orange curve: Elastic Net (AUC=77.075), green curve: Lasso (AUC=74.433), Red Curve: Random Forest (AUC=74.384), purple curve: Ridge Classifier (AUC=78.485), and the brown curve: XG Boost (AUC=74.684).

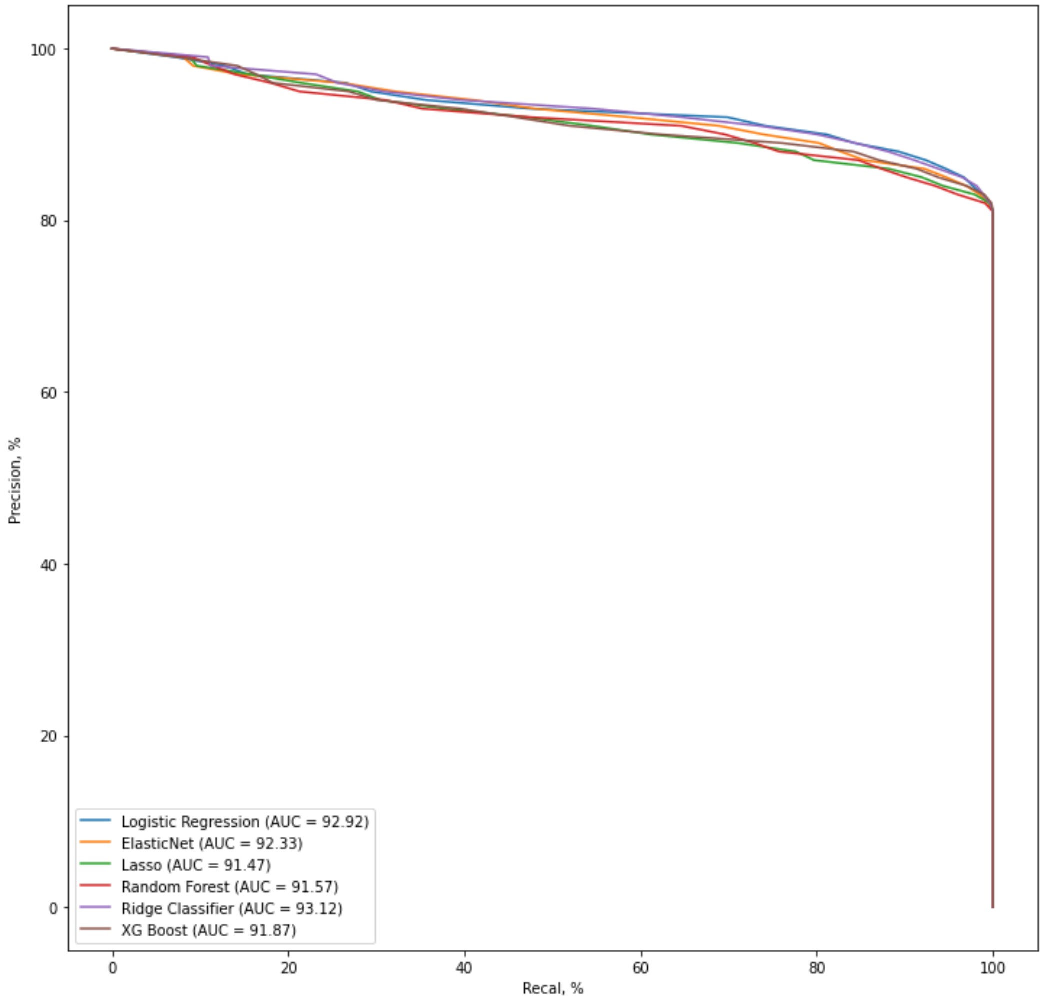

Figure 2. Comparison of the precision-recall curves of all models including feature set 1. Blue curve: Logistic Regression (AUC =92.92), orange curve: Elastic Net (AUC=92.33), green curve: Lasso (AUC=91.47), Red Curve: Random Forest (AUC=91.57), purple curve: Ridge Classifier (AUC=93.12), and the brown curve: XG Boost (AUC=91.87).

(2) Feature set 1: Text with biomedical concepts extracted from MetaMap

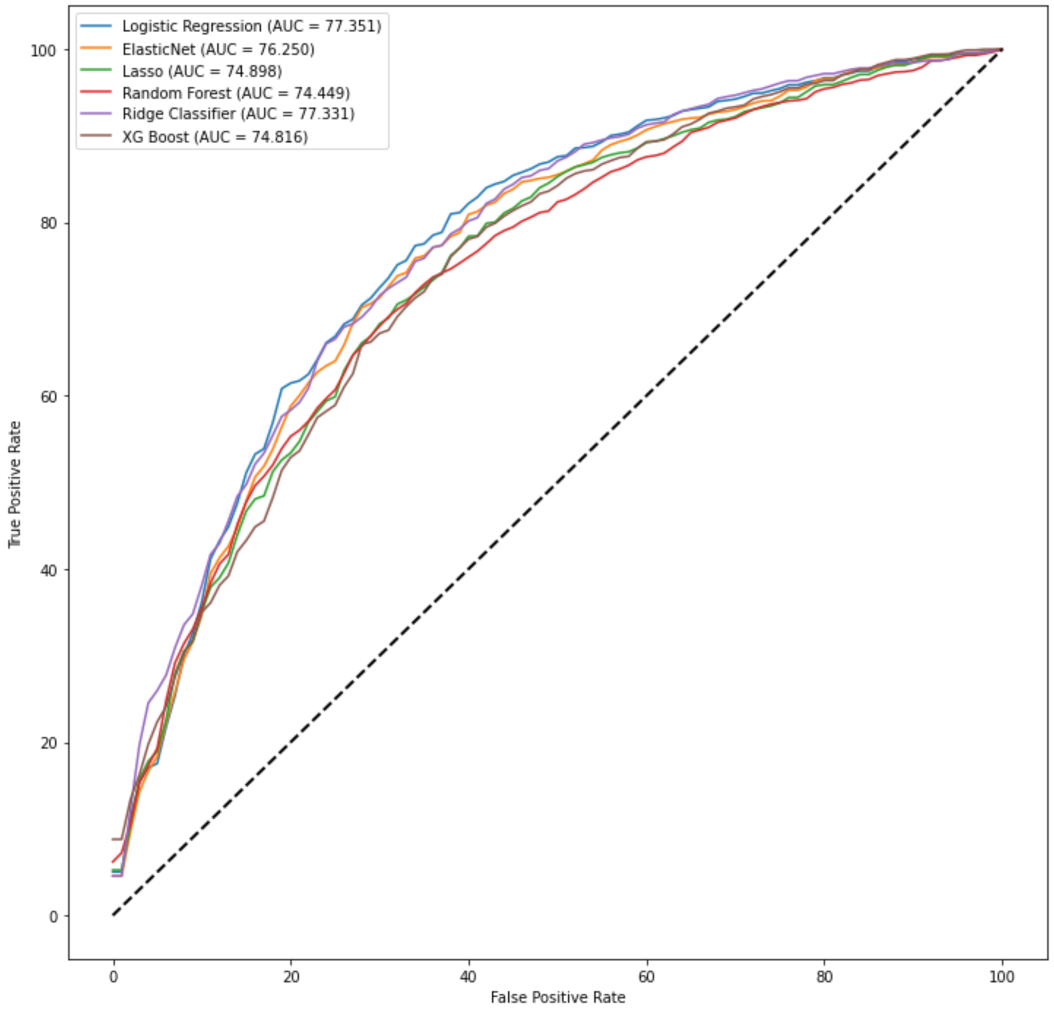

Figure 3. ROC plot shows the performance comparison among six classifiers including feature set 1. Blue curve: Logistic Regression (AUC =77.351), orange curve: Elastic Net (AUC=76.250), green curve: Lasso (AUC=74.898), Red Curve: Random Forest (AUC=74.449), purple curve: Ridge Classifier (AUC=77.331), and the brown curve: XG Boost (AUC=74.816).

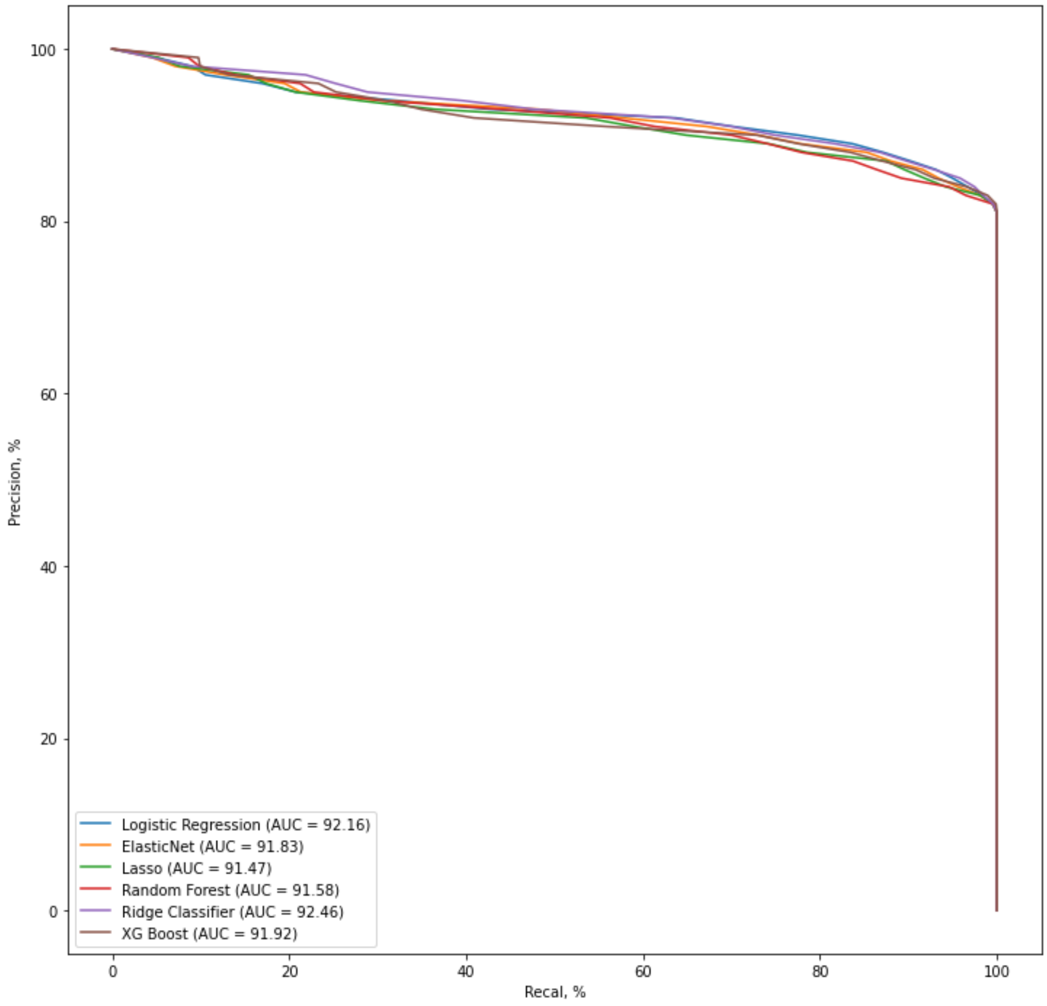

Figure 4. Comparison of the precision-recall curves of all models including feature set 1. Blue curve: Logistic Regression (AUC =92.16), orange curve: Elastic Net (AUC=91.83), green curve: Lasso (AUC=91.47), Red Curve: Random Forest (AUC=91.58), purple curve: Ridge Classifier (AUC=92.46), and the brown curve: XG Boost (AUC=91.92).

(3) Feature set 2: Text with topics and duration of treatment

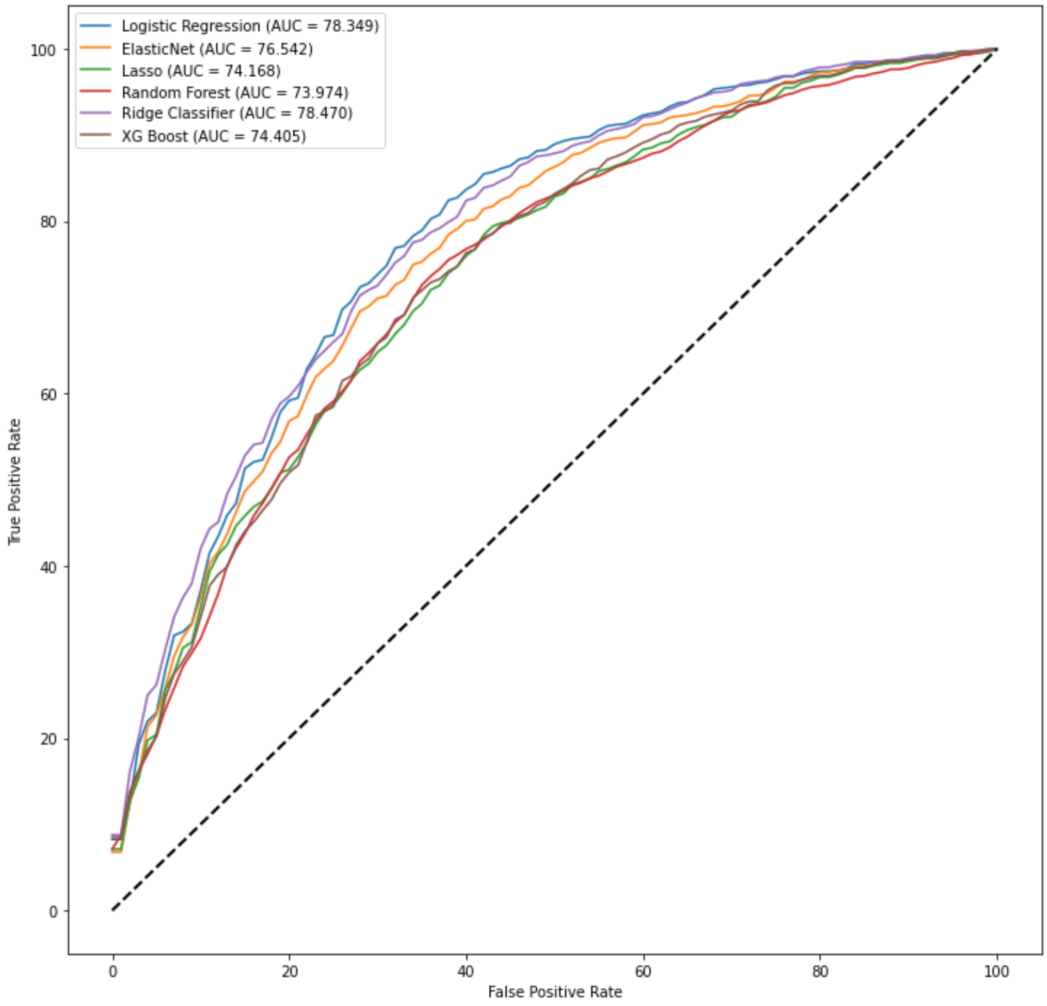

Figure 5. ROC plot shows the performance comparison among six classifiers including feature set 2. Blue curve: Logistic Regression (AUC =78.349), orange curve: Elastic Net (AUC=76.542), green curve: Lasso (AUC=74.168), Red Curve: Random Forest (AUC=73.974), purple curve: Ridge Classifier (AUC=78.470), and the brown curve: XG Boost (AUC=74.405).

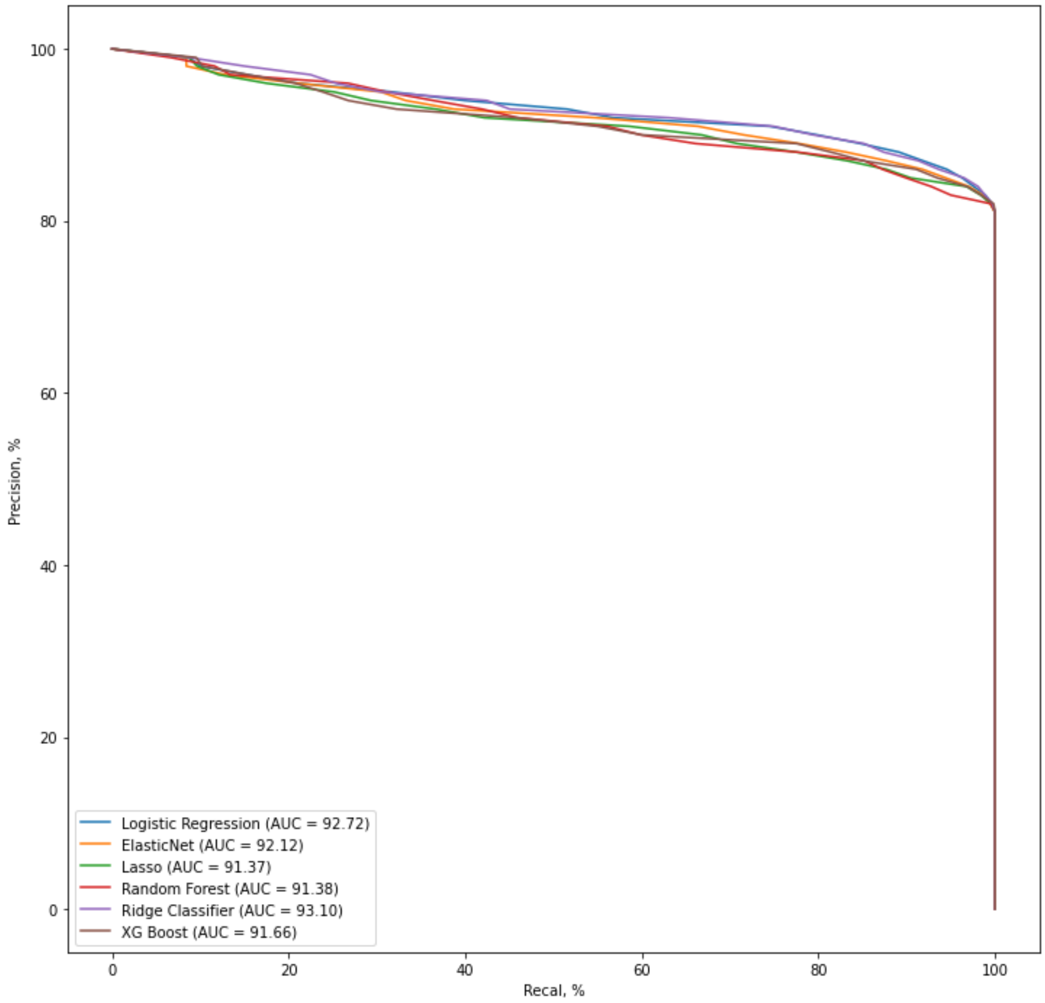

Figure 6. Comparison of the precision-recall curves of all models including feature set 2. Blue curve: Logistic Regression (AUC =92.72), orange curve: Elastic Net (AUC=92.12), green curve: Lasso (AUC=91.37), Red Curve: Random Forest (AUC=91.38), purple curve: Ridge Classifier (AUC=98.10), and the brown curve: XG Boost (AUC=91.66).

(4) Feature set 3: Text with biomedical concepts extracted from MetaMap, topics, and duration of treatment

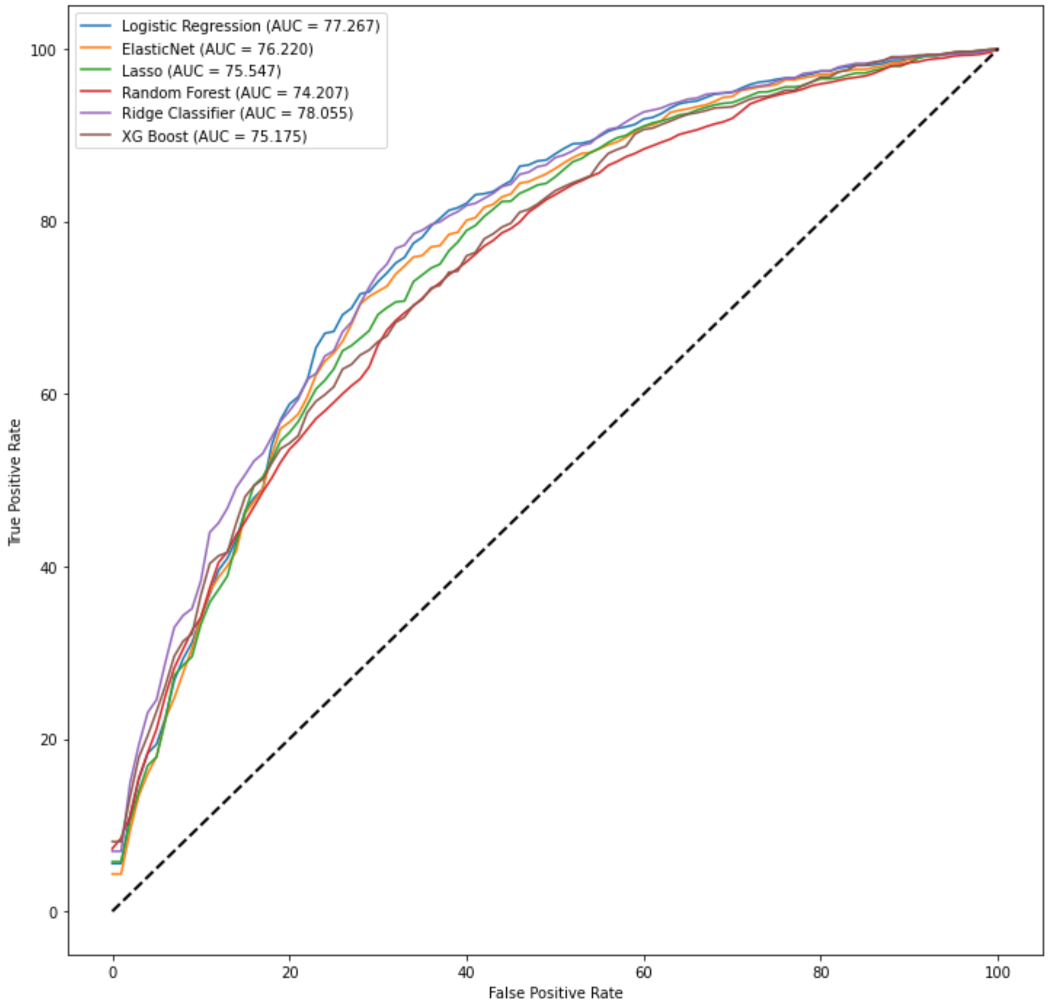

Figure 7. ROC plot shows the performance comparison among six classifiers, including feature set 3. Blue curve: Logistic Regression (AUC =77.267), orange curve: Elastic Net (AUC=76.220), green curve: Lasso (AUC=75.547), Red Curve: Random Forest (AUC=74.207), purple curve: Ridge Classifier (AUC=78.055), and the brown curve: XG Boost (AUC=75.175).

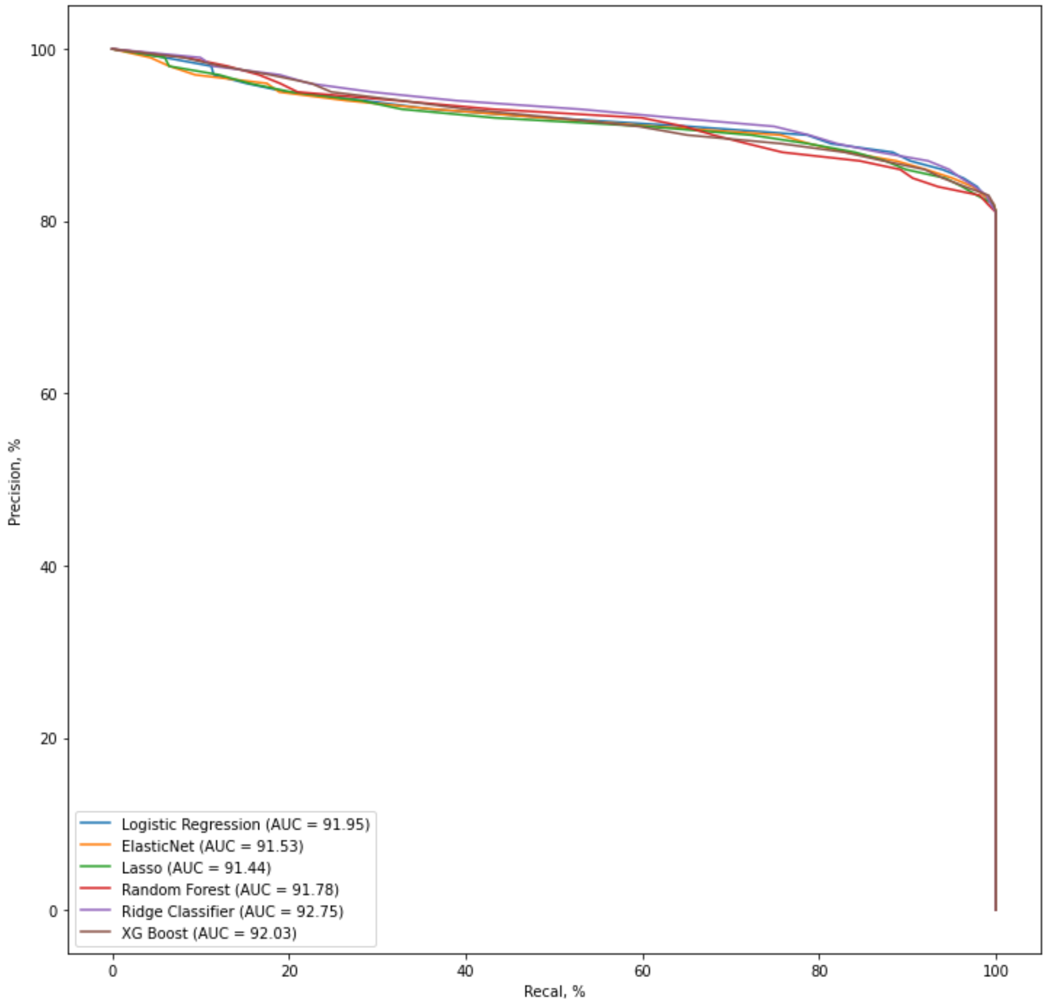

Figure 8. Comparison of the precision-recall curves of all models including feature set 3. Blue curve: Logistic Regression (AUC =91.95), orange curve: Elastic Net (AUC=91.53), green curve: Lasso (AUC=91.44), Red Curve: Random Forest (AUC=91.78), purple curve: Ridge Classifier (AUC=92.75), and the brown curve: XG Boost (AUC=92.03).
